# Improved persistence to statin therapy through a patient counseling intervention in community pharmacies – A nationwide cohort study

**DOI:** 10.1016/j.rcsop.2025.100699

**Published:** 2025-12-22

**Authors:** Karin Svensberg, Björn Wettermark, Jenna Ramsin Eklund, Mohammadhossein Hajiebrahimi, Marie Ekenberg, Albin Tranberg, Sofia Kälvemark Sporrong

**Affiliations:** a Department of Pharmacy, University of Uppsala, Sweden; b Pharmacy & Pharmacology Center, Faculty of Medicine, Vilnius University, Lithuania

**Keywords:** Medication adherence, Statins, Intervention, Community pharmacy services, Sweden, New medicine service

## Abstract

**Background:**

Poor adherence is a well-known problem for statins, key medicines for reducing cardiovascular morbidity and mortality. Community pharmacy services have been identified as a way to increase adherence. We assessed the effect of motivating counseling in Swedish community pharmacies on treatment persistence in patients starting statin therapy.

**Methods:**

In this cohort study, one-year persistence was evaluated in patients who initiated statin therapy (ATC C10AA) between October 2022 and June 2023 following pharmacy-based counseling, and compared with five age- and sex-matched controls per patient from pharmacies not providing the service. Data were collected from Swedish national health registers on dispensed medications, diagnoses and socioeconomic characteristics of patients. Odds ratios for being persistent with 95 % confidence intervals were calculated using a logistic regression model adjusted for socioeconomics, cardiovascular comorbidity and pharmacy size.

**Results:**

A total of 902 patients who had data available in the Swedish national registers received the intervention. They had a higher education and income, mostly Swedish born and they had less history of cardiovascular disease, compared to the 4510 age- and sex-matched controls. The one-year persistence was significantly higher among those who received the service compared to controls (80.2 % compared to 73.6 %). Adjusted odds ratios for being persistent after the intervention was 1.43 (95 % CI 1.19–1.71).

**Conclusion:**

Patients who receive a motivating counseling service in community pharmacies have a higher persistence to statin treatment, one year after initiation, after adjustment for differences in patient characteristics.

## Highlights

• A motivating counseling service in community pharmacies improves statin adherence.
• Targeting low-persistence groups can yield even greater intervention effects.
• Robust register data strengthens valid results on one-year statin persistence rates.

## 1 Introduction

Poor medication adherence is a widespread problem negatively impacting life expectancy and quality of life for millions of people. It has been estimated that almost 200,000 deaths annually in Europe could be avoided if people took medicines as prescribed by their physician.^1^ The economic consequences are also considerable, and in 2011 it was projected that non-adherence was responsible for €80–125 billion of potentially preventable direct and indirect costs in the European Union (EU).^1^ In the report “Evidence for Action”, the World Health Organization (WHO) estimated that only around 50 % of patients with chronic diseases take their medicines as intended, but with a large variation between different settings, diseases and medicines.^2^ One of the areas with the largest potential for improvement is medicines used for preventive purposes including lipid lowering agents.^3^^,^^4^ These medicines are important for preventing cardiovascular morbidity and death, the leading cause of death globally.^5^^,^^6^ Several large randomized clinical trials (RCT) have shown that the risk of infarction and death is reduced by about one-third with statin treatment.^7^ The absolute risk reduction is highest in patients with a high cardiovascular risk, including those with a history of myocardial infarction, stroke or diabetes. However, despite the benefits, several studies have shown both problems of physicians not prescribing and patients not taking their medicines.^8^ Poor adherence may occur in different parts of the chain from prescription to use, and recent adherence research distinguish between initiation (if the patient starts taking the prescribed medications), implementation (the degree to which they comply with the prescription in terms of dose and timing) and persistence (how long the patient continues to take their medications).^9^ Studies with statins have shown problems with all these three components of adherence.^10^^,^^11^ A large Swedish report published ten years ago showed that 27 % of those initiated on therapy had dis-continued their medication one year later.^12^

The reasons behind non-adherence are complex and include both lack of motivation and practical obstacles. Many patients need individualized support to strengthen their knowledge and skills to handle their medications and thus to achieve a high adherence.^2^^,^^13^ Community pharmacies play an important role by providing support and advice that can improve medicine use, including patient adherence.^14^^,^^15^ Therefore, in many countries, a key focus is on services specifically designed to enhance adherence. In England, there has been a structured community pharmacist-led service for many years, *the New Medicine Service* (NMS).^16^ This service, includes two consultations, which aims to increase adherence to medicines in long-term treatment. This is done by reducing problems that arise with newly prescribed medications for patients on long-term treatment, who can quickly become non-adherent.^16^ Similar setups for NMS services are found in Denmark,^17^; Norway^18^; Belgium^19^; France^20^; Poland^21^^,^^22^; various states in the USA^23^; and South Africa.^24^ Studies have evaluated the effects of the NMS and comparable national pharmacy services, showing that the service improves adherence in the short term,^17^^,^^18^^,^^21^^,^^25^ up to 18 weeks (4.5 months). Moreover, for certain medication groups and users (i.e. statin users), the effect has been shown to last up to one year after the intervention.^18^ The service appears to help identify and resolve medication-related problems.^17^^,^^26^ Additionally, both health economic studies from England and a Norwegian report indicate that the service is likely to be cost-effective.^25^^,^^27^, ^28^, ^29^ Hence, these kinds of services seem to work well, however there is a shortage of long-term follow-up studies, based on register (as opposed to self-reported) data.^17^^,^^18^^,^^21^^,^^25^^,^^26^

## 2 Aim

To assess the effect of a motivating counseling service in community pharmacies on one-year persistence to treatment after initiation of statins.

## 3 Methods

In this cohort study using data from Swedish national health registries, we compared one-year persistence to statins for all patients receiving a community pharmacy-based service, including two motivating consultations, with a matched control group.

### 3.1 The setting

In Sweden, there are about 1400 brick-and-mortar pharmacies and seven online pharmacies, primarily owned by four national chains (less than 50 are independent pharmacies).^30^^,^^31^ In 2020, the Swedish government gave the Swedish Pharmaceutical Benefits Agency (TLV) a commission to explore new services in pharmacies to improve quality of care for patients with chronic diseases.^32^ One service tested was an NMS-inspired service, including structured motivating counseling in pharmacies to patients claiming their first prescription of the lipid-lowering medicine statins, with the ambition to improve medication adherence. The service was carried out between October 2022–June 2023 at approximately 130 community pharmacies across the country.^33^ Pharmacies were recruited to meet a national target of 5000 patients across 150 sites. First, a number of pharmacies were distributed to chains/independents as a ratio of their market share. Secondly, recruitment was coordinated by the pharmacy owners (pharmacy chains/independents), with pharmacies volunteering to participate. Pharmacies registering interest were then assessed by the chains. Selection was not random but based on feasibility and heterogeneity criteria such as stable staffing, no planned refurbishments, and ensuring variation in pharmacy type and location (e.g. shopping malls and primary care centers). The final sample included pharmacies from all major chains, one online pharmacy, and two independents.

### 3.2 Intervention - service design

The service design was developed prior to and independently from this evaluation, by a project group within the Swedish pharmacy sector, together with expert functions, external stakeholders and by international experience.^33^ The service was designed to provide support to those who claim their first prescription of a statin. It primarily supported motivation to take the medicine, and practical handling of the medicine in everyday life, in accordance with Horne et al.^34^ No established communication framework was used. However, the service consisted of two counseling consultations, emphasizing active listening, using for example open-ended questions, encouragements, and summaries to motivate patients. The first consultation aimed to, through open dialogue, explore patients' perspectives, beliefs, and understanding regarding using statins. Pharmacists were instructed to use a motivating approach to address concerns, correct misunderstandings, and reinforce adherence-promoting behaviors. Based on this, the pharmacist was expected to provide customized advice and information to simplify the use of the medication, and confirm or increase the patient's intrinsic motivation to take the medicine as prescribed by the physician. No additional materials such as dashboards, scheduling aids, calendars, or patient trackers for monitoring side effects or adherence were used. Intervention pharmacists used structured documentation cards (see Appendix 1). These documents outlined examples of key questions and the three phases of the consultation (understanding the patient's motivation, practical use of the medicine, closing and summarizing of the conversation) functioning both as a documentation tool and as a guide to support consistency in the intervention delivery. After the consultation, the patient received documentation from the consultation (see Appendix 1), the pharmacist also documented (on the documentation card) the consultation to support follow-up during a second consultation. The document was included in the pharmacists' training, and service adherence was monitored as part of the pharmacies' internal quality assurance for the service.^33^ The first consultation took place immediately after the prescription was filled. The second consultation was conducted 2–4 weeks after the first, and aimed to follow up on the first consultation, as well as investigate the patient's experience after having used the medicine for a number of weeks.^33^ In the physical pharmacies the first conversation took place in person in the pharmacy, the second either in the pharmacy or per telephone, preferred by the patient. The online pharmacy consultations were performed over phone.

Pharmacists became eligible to provide the service after completing a standardized two-hour theoretical training, covering the service framework, lipid-lowering pharmacotherapy, adherence, communication skills, and effective service delivery. Additionally, they completed two hours of hands-on consultation practice with colleagues. The training package was also developed by the Swedish Pharmacy Association with a working group of experts from their member companies, and a consultant specialized in communication training of pharmacists. The training built on international implementation experience and adherence research.^34^ This education aimed to ensure that the consultations and recruitment of participants were carried out in a standardized way.

### 3.3 Study design and population

The inclusion criteria for patients participating in the interventions were: the patient was 18 years or older, had a new prescription for a statin, had not received a statin prescription in the previous 12 months, and that the medicine was not picked up by someone else. Participating pharmacists were instructed to trying to recruit all patients fulfilling the inclusion criteria at each study-site. Identification of eligible patients was carried out manually by the pharmacist. In this process pharmacists were expected to explain the service to the patient and in addition inform them about this study, and to obtain verbal consent to participate in the study. No extra resources or incentives were provided.

The effect of the intervention was assessed using a cohort study design with individual level data on dispensed prescriptions, diagnoses, and socioeconomic characteristics, linked by the National Board of Health and Welfare (NBHW), through each patient's unique personal number.^35^ All people in the country who claimed their first prescription with statins (ATC code C10AA) during the period October 2022 to June 2023 were included. Patients who did not claim any statin prescription during one year prior to this period (wash-out period) were counted as patients starting on therapy. Patients who received the full intervention, that is both counseling consultations, constituted the intervention group. As control group, five age- and sex-matched controls were identified for each person who received the intervention (hereafter named “matched controls”). The choice of five controls is rather common in non-randomized intervention studies and it was selected as a trade-off between minimizing bias and maximizing the sample size and power of the study.^36^ All controls started statin treatment during the same period, but were dispensed their initial prescription from pharmacies that did not participate in the intervention (offering the service). In addition, comparisons were made with a non-intervention group consisting of patients who visited the participating pharmacies without receiving the full intervention with two motivating counseling consultations (hereafter named “non-intervention group”).

### 3.4 Data sources

All data were collected from Swedish national health registers held by NBHW, and Statistics Sweden. The registers used were the Swedish National Prescribed Drug Register (SPDR),^37^ the National Patient Register (NPR),^38^ the National Cause-of-Death Register (NCDR),^39^ and the Longitudinal Integration Database for Health Insurance and Labour Market Studies (LISA).^40^ Aggregated service-delivery data from the Swedish Pharmacy Association were used to determine the number of patients invited to the service and how many received only one consultation. Individual-level data were available only for patients who completed the full service, since after both consultations had taken place, pharmacists submitted an electronic form to the research team containing the patient's personal identification number. These identifiers were transferred to the NBHW to flag individuals who received the intervention and identified (by data from the Swedish eHealth Agency) the pharmacies where it was delivered. All data were pseudonymised before made available to the research team.

The SPDR includes detailed information on all prescribed medications dispensed in Swedish pharmacies since July 2005, including medication names, substances, dosages, dates of prescribing and dispensing, quantities dispensed and information about the prescriber. Patient information such as age, sex, personal identification number and other demographic details was obtained through this register, in addition to information on statin dispensation (ATC: C10AA) and previous dispensing of antidiabetic (ATC: A10), antithrombotic (ATC: B01), and antihypertensive (ATC: C03, C07, C08, and C09) agents. Through the NPR, information was obtained about patients' medical history and previous diagnoses recorded during hospitalization or outpatient hospital consultations, registered according to ICD-10 (10th version of the International Classification of Diseases).^38^ The diagnoses included in the study were myocardial infarction (MI) – (ICD: I21, I22, I24.1, I25.2), ischemic heart disease (IHD) – (ICD: I20, I24 excluding I24.1, I25 excluding I25.2–4, stroke/TIA (ICD: G45, G46, I63-I66, I69.3–4), and peripheral artery disease (PAD) (ICD: I70, I71, I73.9, I74, K55). These diseases were used to identify patients who received the statin medication for secondary prevention. All of the diagnoses above were recorded at index date (date of first statin dispensing) and up to three years prior. The NCDR was used to register potential deaths during the study period.^39^ The LISA-register authorized by the Statistics Sweden (SCB) contains sociodemographic data over the Swedish adult population aged ≥15 years and was used to obtain information on education level, marital status, income and country of birth for all patients.^40^ The education levels were categorized into three groups: Primary school (≤ 9 years of education), Secondary school (10–12 years), and Academic (>12 years). Marital status was divided into two groups, married or unmarried. The country of birth was defined as one of the following subgroups: Sweden, Nordic countries excluding Sweden, Europe (EU28) excluding Nordic countries and out of EU28. Time-dependent information was collected from up to one year prior to the index date.

### 3.5 Outcome measure and covariates

The primary endpoint was persistence (the proportion that remained on statin treatment after one year), defined as dispensed prescription in the time window of 10–14 months after initiation, a commonly used method called the “anniversary method”.^41^ In the analysis, persistence to treatment one year later was compared between the intervention group and the matched controls, and to the non-intervention group respectively. We also describe age, sex, sociodemographic characteristics, and cardiovascular morbidity in the intervention group, compared to the matched controls and the non-intervention group.

### 3.6 Statistical analysis

Descriptive statistics including mean and interquartile range (IQR), number and percentage of categorical variables were used to present sociodemographic characteristics, cardiovascular morbidity and previous use of other cardiovascular- or diabetes medications in the intervention and control groups. Absolute difference in persistence was calculated in total and stratified based on sociodemographic characteristics and comorbidities. In addition, a logistic regression model was utilized to address imbalances between the intervention and control groups. Odds ratios for being persistent were calculated for the intervention, stepwise adjusted for a) socioeconomics (*model 1*), b) socioeconomics + cardiovascular comorbidity (secondary or primary prevention) *(model 2*), and c) socioeconomics + cardiovascular comorbidity + pharmacy size (*model 3*). All analyses were performed using the SAS software, version 9.4.

### 3.7 Ethical considerations

The study was approved by the Ethical Review Board (registration number 2022–03910-01). All intervention participants received written and verbal study details and provided informed consent. Data were securely stored and digitally encrypted. All patient data accessed by the researchers were fully pseudonymized. Consequently, it was not possible to identify individual patients or healthcare providers.

## 4 Results

### 4.1 Pharmacy participation and patient recruitment

Out of 141 pharmacies that signed up, 127 (90.1 %) conducted the intervention (i.e. two consultations) to at least one patient and hence participated in the intervention. From October 2022 to June 2023, these pharmacies had 16,006 new statin users who claimed their first prescription (out of 121,456 persons claiming their first statin prescription in all Swedish pharmacies).

**Fig. 1:**
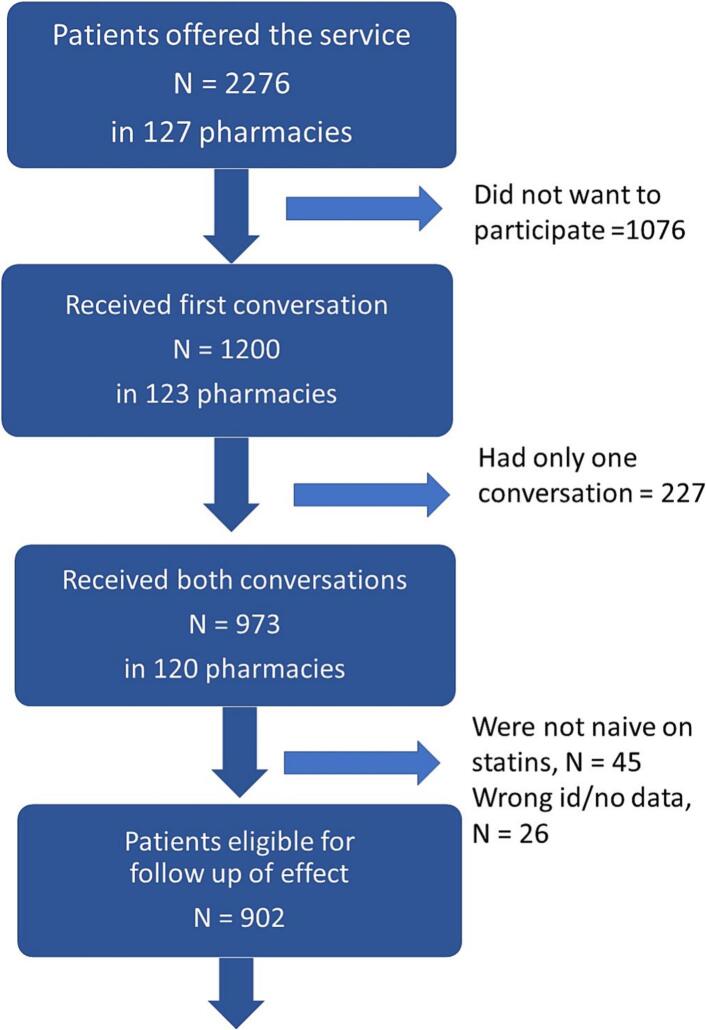
Flow-chart presenting the recruitment of patients to motivating consultations in pharmacies.

In total, 2276 were invited to participate, 227 had only one conversation, and 973 (43 %) received the intervention (i.e. both consultations) (Fig. 1). After excluding 45 non-naïve patients (statin dispensed during the wash-out period) and 26 with identifier errors, 902 were assessed. These patients represented 5.6 % of statin starters at the intervention pharmacies and 0.8 % of all starters in any pharmacy in the country. To assess the effect on persistence, we compared patients receiving both consultations with the matched controls visiting pharmacies not participating in the intervention.

**Fig. 2:**
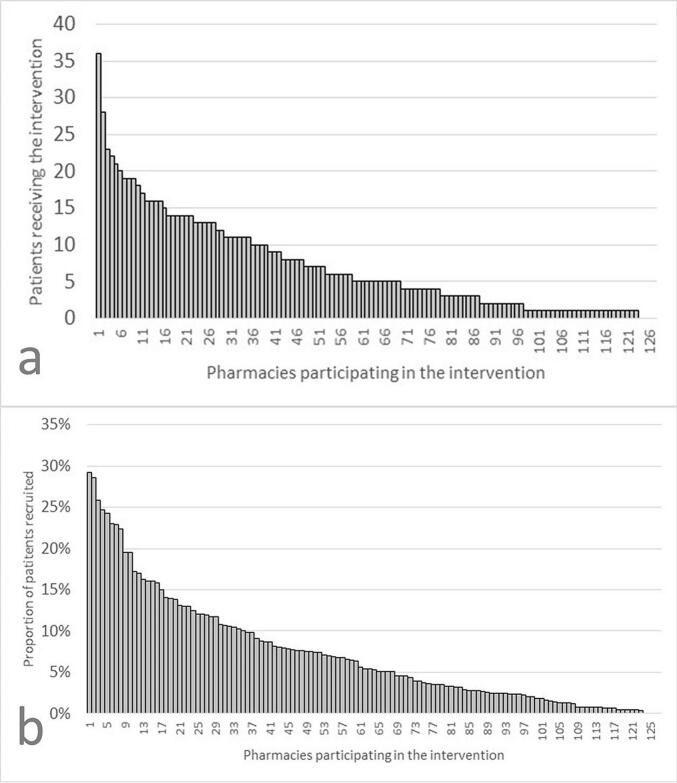
Recruitment to the study by intervention pharmacy Oct 2022- June 2023. a) number of patients who received the complete intervention with two consultations per pharmacy, b) Proportion of patients who during the period claimed their first statin prescription, was included in the study and received two motivating consultations.

Pharmacies varied widely in size and demographics, with eligible patient numbers ranging from 22 to 309. The percentage of patients offered and accepting the service ranged from 0 to nearly 100 %. At the 127 participating pharmacies, the number receiving both motivating consultations varied from 0 to 36, with one-fifth conducting the intervention with only one patient (Fig. 2a). The proportion of all patients who visited the pharmacy to claim their first prescription for statins, who were included in the intervention and participated in both consultations, varied between 0 and 29 % (Fig. 2b).

### 4.2 Demographics for participating patients and controls

Those who received the full intervention with two consultations were to a larger extent female, people with higher education and born in Sweden, compared to those who visited the same pharmacy and did not participate (Table 1). There were also fewer younger people, fewer with low income, and fewer with previous cardiovascular disease (secondary prevention) who completed the study and received the two counseling consultations. However, the pharmacies that offered the service were relatively comparable to the pharmacies that did not participate in the study. After matching for age and sex, the intervention patients still differed from the controls in terms of educational level, county of birth, income and cardiovascular comorbidity (Table 1). Two thirds of all intervention patients and a similar proportion of controls were previously treated with antihypertensives. Around one fifth previously received antidiabetics. The most commonly prescribed statin for initiation was atorvastatin (70 % of those who received both consultations) followed by rosuvastatin (28 %). The distribution between statin substances was similar to the matched controls and non-intervention group, respectively.

**Table 1:** Comparison between patients who received both consultations and a) those who did not receive both consultations in the pharmacies that participated in the study and offered the service b) age- and gender-matching controls from pharmacies that did not offer the services.

	Intervention group (902)		Non-intervention group (a)⁎ (15,104)		Matched controls from non-participating pharmacies (b) (4510)	
	N	%	N	%	N	%
Sex						
Men	433	48.0	8176	54.1	2165	48.0
Women	469	52.0	6928	45.9	2345	52.0
Age						
<55	150	16.6	3258	21.6	741	16.4
55–64	228	25.3	3904	25.9	1134	25.1
65–74	303	33.6	4318	28.6	1540	34.2
≥75	221	24.5	3624	24.0	1095	24.3
Mean (±SD)	65.3 (±11,9)		64.4 (±12.9)		65.3 (±11,9)	
Median (IQR)	67 (58–74)		65 (56–74)		67 (58–74)	
Range	22–92		18–100		21–92	
Level of education						
Primary school (≤9 years)	131	14.5	2774	18.4	864	19.2
Secondary school (10–12 years)	418	46.3	6837	45.3	2022	44.8
Academic (>12 years)	353	39.1	5493	36.4	1624	36.0
Marital status						
Married	500	55.4	8074	53.7	2343	52.2
Unmarried	402	44.6	6956	46.3	2148	47.8
Missing	0		74		19	
Income (SEK)						
<181,900	175	19.4	3479	23.2	1153	25.7
181,900-261,300	222	24.6	3695	24.6	1120	24.9
261,400-382,000	264	29.3	3902	26.0	1115	24.8
>382,000	241	26.7	3954	26.3	1103	24.6
Missing	0		74		19	
Country of birth						
Sweden	794	88.0	12,110	80.2	3521	78.1
Nordic countries except Sweden	24	2.7	550	3.6	180	4.0
EU28 outside the Nordic countries	19	2.1	498	3.3	161	3.6
Outside the EU28	65	7.2	1941	12.9	648	14.4
Missing	0		5		0	
Comorbidity						
Myocardial infarction	24	2.7	1000	6.6	291	6.5
Stroke/TIA	50	5.5	1445	9.6	440	9.8
Ischemic heart disease	36	4.0	1038	6.9	284	6.3
Peripheral vascular disease	20	2.2	436	2.9	112	2.5
Prevention status						
Secondary prevention	111	12.3	3095	20.5	907	20.1
Primary Prevention	791	87.7	12,009	79.5	3603	79.9
Other medicines						
Antihypertensives	571	63.3	9785	64.8	2961	65.6
Antidiabetic agents	180	20.0	3270	21.7	917	20.3
Antithrombotics	88	9.8	1614	10.7	505	11.2

⁎ 227 (1.5 %) of these received only one conversation, and thus did not complete the intervention. Information was not available to identify this group and thus exclude them from the analysis.

### 4.3 Persistence - proportion remaining on statins one year later

The proportion of patients who continued to claim their statin prescriptions after one year was significantly higher among those who received two counseling consultations compared to the matched controls (80.2 % compared to 73.6 %) (Table 2). The crude difference in persistence between the intervention group and the non-intervention group was of the same magnitude (80.2 % compared to 72.8 %) (Appendix 2.). Significant differences were observed for most subgroups, when comparing intervention patients with the matched controls and the non-intervention group (Table 2; Appendix 2). People dying during follow-up have not been excluded in the analysis. During the first year after initiation of statin therapy, 8 people (0.9 %) died in the intervention group and 72 people (1.6 %) in the matched control group. The difference was not significant and is probably explained by the higher cardiovascular comorbidity in the control group.

**Table 2:** Comparison of persistence after one year between patients who received both consultations (intervention) and age- and sex-matched control patients from pharmacies who did not offer the services All results are stratified, but unadjusted.

	Intervention (n = 902)			Matched Controls (n = 4510)			Difference (* = significant)		
	Total	Persistent		Total	Persistent		%-units	95 % C.I.	
	**No.**	**No.**	**%**	**No.**	**No.**	**%**			
TOTAL	902	723	80.2	4510	3319	73.6	6.6	(3.7–9.5)	*
Sex									
Men	433	341	78.8	2165	1594	73.6	5.2	(0.9–9.4)	*
Women	469	382	81.5	2345	1725	73.6	7.9	(3.9–11.8)	*
Age									
<55	150	104	69.3	741	491	66.3	3.0	(−5.1;11.2)	
55–64	228	183	80.3	1134	837	73.8	6.5	(0.7–12.2)	*
65–74	303	249	82.2	1540	1173	76.2	6.0	(1.2–10.8)	*
≥75	221	187	84.6	1095	818	74.7	9.9	(4.5–15.3)	*
Level of education									
Primary school (≤9 years)	131	105	80.2	864	625	72.3	7.9	(0.4–15.3)	*
Secondary school (10–12 years)	418	339	81.1	2022	1509	74.6	6.5	(2.3–10.7)	*
Academic (>12 years)	353	279	79.0	1624	1185	73.0	6.1	(1.3–10.8)	*
Marital status									
Married	500	416	83.2	2343	1746	74.5	8.7	(5.0–12.4)	*
Unmarried	402	307	76.4	2148	1566	72.9	3.5	(−1.1;8.0)	
Income (SEK)									
≤181,800	175	135	77.1	1153	805	69.8	7.3	(0.6–14.1)	*
181,900-261,300	222	180	81.1	1120	827	73.8	7.3	(1.5–13.0)	*
261,400-382,000	264	212	80.3	1115	848	76.1	4.2	(−1.2–9.7)	
>382,000	241	196	81.3	1103	832	75.4	5.9	(0.4–11.4)	*
Country of birth									
Sweden	794	648	81.6	3521	2668	75.8	5.8	(2.8–8.9)	*
Nordic countries excl Sweden	24	22	91.7	180	125	69.4	22.3	(9.3–35.2)	*
EU28 excl Nordic countries	19	14	73.7	161	104	64.6	9.1	(−12.0; 30.2)	
Outside the EU28	65	39	60.0	648	422	65.1	−5.1	(−17.6;7.3)	
Prevention status									
Secondary prevention	111	90	81.1	907	715	78.8	2.3	(−5.5;10.0)	
Primary prevention	791	633	80.0	3603	2604	72.3	7.7	(4.6–10.9)	*

95 % confidence interval.

### 4.4 Effect of the intervention

**Fig. 3:**
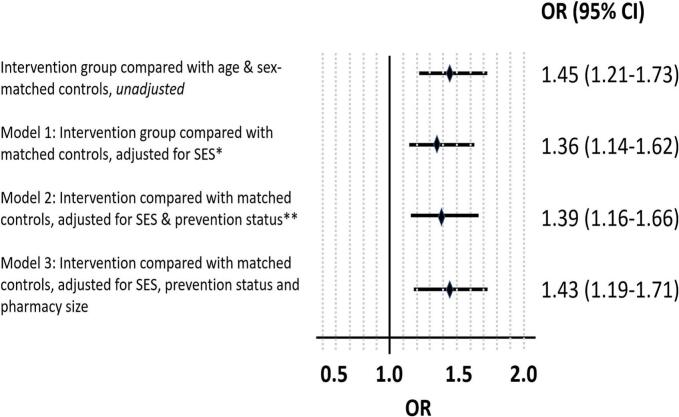
Odds ratios with 95% confidence intervals of being persistent one year later in people who received both consultations (intervention) compared to the age- and sex-matched controls. Unadjusted and adjusted for differences in patient composition. *Socioeconomics (SES) = level of education, marital status, income and country of birth. **Prevention status = been prescribed statin as secondary prevention, i.e. previously had either myocardial infarction, ischemic heart disease, stroke/TIA and/or peripheral vascular disease.

The effect of the intervention was analyzed by calculating odds ratios for being persistent, adjusting for the differences in patient characteristics between those who received both consultations and the matched controls. A significant difference in persistence was observed, which remained after adjusting for socioeconomics, cardiovascular morbidity and pharmacy size. The adjusted odds ratios for persistence after the intervention was 1.43 (95 % CI 1.19–1.71) (Fig. 3).

## 5 Discussion and conclusion

### 5.1 Discussion

In this prospective cohort study, assessing if structured motivating counseling service in pharmacies could improve medication adherence, we found a significantly higher proportion of people initiated on statin therapy who remain on therapy one year later among patients receiving the intervention compared to their age and sex-matched controls. This difference remained significant after adjusting for differences between the two groups in socioeconomic characteristics, cardiovascular morbidity, and pharmacy size.

In the intervention group, the persistence was 80 %, a high figure compared to previous studies assessing persistence to statin therapy.^3^^,^^10^^,^^11^ It is difficult to compare persistence rates between different studies as the populations, follow-up time, and method to assess persistence differ substantially. In a review by Cramer et al.,^3^ one-year persistence to lipid-lowering medicines varied between 35 and 85 % across studies, with an average of 66 %. In the comprehensive Swedish national report (2013), on utilization of statins, 73 % of patients who were initiated on statin treatment were persistent one year later.^12^ This figure is the same as we found in the matched control group. A systematic review of 49 studies published between 2000 and 2018 reports that six different types of interventions have been shown to be effective in improving adherence.^42^ These include patient education, improved treatment regimen, clinical pharmacist, cognitive behavioral therapy, reminders, and financial incentives. The intervention evaluated in this paper contains elements similar to other interventions with education and motivational interviewing, that previously have shown a positive effect. It has also been confirmed by a systematic review of 771 intervention studies that such interventions are effective in community pharmacies.^43^

Our study is unique in examining the one-year persistence based on robust data from high-quality registers,^35^^,^^38^^,^^44^^,^^53^ while most previous research have focused on shorter follow-up periods and relied on self-reported adherence scales.^17^^,^^18^^,^^21^^,^^25^^,^^28^ Only one previous Norwegian study and one study from the USA on NMS services have examined long-term statin adherence, and they report similar results to ours.^18^^,^^45^ In the Norwegian study the statin users in the intervention group had an MPR (Medication Possession Ratio) based adherence rate of 66.5 % compared to 57.4 % in a control group, after 52 weeks.^18^

In routine Swedish community pharmacy practice, dispensing encounters last around 3–4 min, with counseling typically limited to 10–25 s.^46^ In the intervention, pharmacists instead conducted structured conversations addressing the patient's motivation, concerns, and practical management of the new treatment. Elements that are not always part of usual practice. Although we do not have site-specific timing data, pharmacists reported in a survey that each consultation generally took 5–10 min.^47^ Some variation between sites is likely, but feedback indicated that all pharmacies spent more time on these consultations compared with routine encounters, which is likely one explanation for the positive persistence outcomes observed in this and other similar studies.^17^^,^^18^^,^^21^^,^^25^^,^^28^ It has been reported that patients tend to forget half of the medical information they receive from well-intending healthcare professionals, with considerable variation — sometimes as little as 20 % retention.^48^ The timing, information structure, and communication skills focusing on motivation are key factors in internalizing medical information. Our positive results suggest that motivating counseling at the point of dispensing a new medication is a favorable setting. It can help reinforce, repeat, and allow patients to better process the information received from the hospital/primary care. This approach could also serve as both an extension and easing the burden of already strained healthcare services. In both Norway and the UK, this service has been suggested as cost-effective for the health care system.^25^^,^^27^, ^28^, ^29^

The results showed that the intervention group differed from the matched controls regarding some sociodemographic factors, and probably, on group level, had higher health literacy. There may be several reasons why fewer patients with low health literacy received the intervention, such as language barriers. This is, however, not optimal, as previous research has shown that adherence to statin therapy is lowest in people with low socioeconomic status.^50^^,^^51^ People with higher health literacy tend to be more adherent, probably due to greater opportunity of active involvement in treatment decisions.^52^ The service's benefit would probably have been even higher if less adherent people/groups had been recruited to a larger degree. Further research should explore whether motivation can be even further enhanced. For example, patients prescribed statins for primary prevention showed a higher persistence rate in the intervention group than in the matched control group—nearly matching the rate observed among those receiving secondary prevention. This is noteworthy, as primary prevention groups typically have lower adherence compared to secondary prevention.^49^ Given that this group can be challenging to motivate, it is promising that a pharmacy service appears to effectively engage this population. By tailoring the service to specific groups, it might be even more effective.

### 5.2 Methodical discussion

This study has many strengths. The intervention was carried out in 127 pharmacies of different size across Sweden, with large differences in patient demography. All data were collected from national Swedish health registers of high quality, and we adjusted for socio-economy and comorbidity, factors that have previously been shown to be associated with adherence and could act as confounders. The SPDR used to assess the main outcome, has 99.7 % coverage of unique identities of patients.^53^ The outcome measure, one-year persistence, was measured with the anniversary method.^41^ Although it provides a rather crude measure of adherence, it is an objective measure, and drug dispensing data is considered the most reliable method for measuring persistence.^9^ No formal fidelity assessment of the service delivery was conducted within this study. However, participating pharmacies completed an internal quality control programme developed by the Swedish Pharmacy Association, which included 16 indicators related to the implementation of the service.^33^ These indicators suggested a high overall adherence.

We acknowledge some important limitations. The target of 5000 customers was based on the fact that around 100,000 patients per year had newly initiated statin treatment in recent years. With 10 % of all pharmacies participating over six months it was estimated that pharmacies would encounter about 5000 eligible customers. However, only 2278 recruitment attempts were made (46 % of the target), and 973 services were completed (65 % of the target). Thus, the main shortfall concerned the number of recruitment attempts. More than half of those approached accepted, resulting in a recruitment rate of 53 %, which is comparable to a similar study.^18^ Pharmacy location data were available, but geographical variation was not analyzed in the present study. Recruitment relied on pharmacists manually identifying eligible patients during routine workflow, which required checking dispensing histories, confirming new statin use, and explaining both the service and the research study. Without technical support or additional resources, this process was probably time-consuming and often constrained by high workload,^47^ leading to missed patients and the possibility of informal selection bias. As most pharmacies had not provided pharmaceutical services for many years, some uncertainty around service delivery may also have contributed to the limited recruitment. Simplifying recruitment procedures will be important for future implementation.

As the participating pharmacies did not randomly allocate patients to receive the intervention, the effect was assessed with a cohort design, which is considered providing the highest evidence for evaluation of interventions when randomization is not feasible.^54^ In this study, we handled confounding through matching for age and sex, and adjusting for socioeconomics, cardiovascular morbidity, and pharmacy size. However, we acknowledge that there may still be important differences between patients who received the intervention and the matched controls, such as lifestyle factors and differences in health literacy. Furthermore, one in five recruited patients received only one consultation, and were not included in the effect analysis. Since only patients who completed both consultations were reported to the research team. A follow-up according to “intention-to-treat” would have been preferred but not possible; however, the restriction is justified as the intervention was only properly implemented with two consultations. In addition, the matched control group was selected from pharmacies without the service, hence there is no risk that it was “contaminated” with people receiving parts of the intervention. The choice of control group also minimized the risk that patients not receiving the intervention were dispensed by pharmacists changing their way of consulting after being educated on how to do motivating counseling. Still, it cannot be excluded that the higher adherence in the intervention group, was explained by a Hawthorne-effect if those who received the intervention changed their behavior just because they were being observed.^55^ Finally, in the study, we did not assess if the higher persistence in the intervention group corresponded to any health benefits for the patients. However, even a minor increase in adherence might add value, given the large number of patients (with high cardiovascular risk) being treated with statins, and several systematic reviews have previously shown that improved adherence to statin therapy leads to reduced mortality and morbidity.^56^, ^57^, ^58^

### 5.3 Conclusion

Patients who receive a motivating counseling service in community pharmacies have a higher persistence to statin treatment, one year after initiation. Consequently, it has the potential to alleviate pressure on already strained healthcare services. By targeting patient groups with low persistence, even greater intervention effects might be achieved.

### 5.4 Practice implications

Pharmacists can significantly increase statin persistence through motivating counseling, with robust data confirming one-year persistence. By focusing on patients with low persistence, pharmacy services could further enhance long-term treatment outcomes.

## CRediT authorship contribution statement

**Karin Svensberg:** Writing – review & editing, Writing – original draft, Methodology, Conceptualization. **Björn Wettermark:** Writing – review & editing, Writing – original draft, Resources, Methodology, Formal analysis, Data curation, Conceptualization. **Jenna Ramsin Eklund:** Writing – review & editing, Project administration, Investigation, Data curation. **Mohammadhossein Hajiebrahimi:** Writing – review & editing, Visualization, Formal analysis, Data curation. **Marie Ekenberg:** Writing – review & editing, Formal analysis. **Albin Tranberg:** Writing – review & editing, Project administration. **Sofia Kälvemark Sporrong:** Writing – review & editing, Writing – original draft, Resources, Methodology, Funding acquisition, Data curation, Conceptualization.

## Declaration of generative AI and AI-assisted technologies in the writing process

During the preparation of this work the author(s) used ChatGPT in order to improve language. After using this tool/service, the author(s) reviewed and edited the content as needed and take(s) full responsibility for the content of the publication.

## Funding

The service was developed by the Swedish Dental and Pharmaceutical Benefits Agency (TLV) in collaboration with the Swedish Pharmacy Association (Sveriges Apoteksförening). This was based on a government assignment to TLV. The study was financed by the Swedish Pharmacy Association, in order to evaluate the service. They had no influence on study design or analysis.

## **Declaration of competing interest**

Declarations of interest: none. The other authors declare that they have no competing interests.

## Data Availability

For confidentiality reasons it is not allowed to publicly share the pseudonymized patient-level data. However, upon a reasonable request, additional analyses can be conducted after contact with the corresponding author.

## Appendix A Supplementary data

Documentation card: consultation 1, 2 and the costumer card Image 1

One-year persistence: intervention vs. non-intervention group in the 127 participating pharmacies. Image 2
