# Supplementary material for: Improved persistence to statin therapy through a patient counseling intervention in community pharmacies – A nationwide cohort study
Source: Explor Res Clin Soc Pharm. 2025 Dec 22;21:100699. doi: 10.1016/j.rcsop.2025.100699 (PMC12811631; doi:10.1016/j.rcsop.2025.100699)
Supplement: Supplementary file 1 — Documentation card: consultation 1, 2 and the costumer card [file mmc1.docx]

**Pharmacy information:**
Pharmacy chain:………………………………… Pharmacy name:………………………………………… **Customer has given verbal consent to participate in the study**

**Customer information**:

Name:……………………………………………… Personal ID number:……………………………………

Email/Postal Address:………………………………………………………………………………………………………………………. Phone Number:……………………….

**Support for pharmacist conversation 1**

Ask questions. Listen and confirm the costumer. Adapt actions based on the customer's response.

**1. Understand the motivation to take the medicine:**

Example of opening questions:

- What do you think about this medicine/treatment?
- What do you gain by starting this medicine?
- Do you have any questions about the medicine or treatment?

Example follow-up questions if the customer expresses concern or low motivation:

- Is there anything that worries you?
- What do you need to feel secure about the treatment?
- What do you think might happen if you don't take the medication?

**2.** **Understand whether the customer feels confident handling the medicine in daily life**
Example of opening questions:

- What information have you received about how to take the medicine?
- How will you make sure to take the medicine every day?

Example follow-up questions if the customer expresses challenges with adherence:

- You mentioned it feels difficult with... what do you think would help?

**3. Summary**
Encourage the customer to summarise what they take with them and what they will do to remain adherent. Write this on the “Customer Card” for the customer, and on the right-hand side for follow-up at Consultation 2.

Example of questions:

- What do you think about what we discussed today?
- What do you think is most important to remember to take the medicine daily?

**Pharmacist's documentation from conversation 1**

**Customer’s motivation to take the medicine:**
The customer is motivated and wants to take the medicine
The customer is hesitant/not motivated and does not want to take the medicine

**Customer’s confidence in handling the medicine in daily life:**
The customer knows how, when, and for how long the medicine should be taken
The customer feels confident handling the medicine in their daily life
The customer expresses challenges in handling the medicine in their daily life

**To follow up in Consultation 2 (tips/advice/reflections/challenges/concerns/other):**

__________________________________________________________________

__________________________________________________________________

__________________________________________________________________

_______________________________________________________

**Consultation 1 completed:**Date: Pharmacist:

**Consultation 2 booked:**
Visit at the pharmacy Phone call Date: Time:

**Support for pharmacist conversation 2**
Ask questions. Listen and confirm. Adapt actions based on the customer's response.

**1. Follow up on the documentation from Consultation 1**
Example opening question:
● *Last time you mentioned… what are your thoughts about that now?*

**2. Understand the motivation to continue taking the medicine**
Example opening questions:
● *How do you experience the treatment now that you have taken the medicine for a while?*
● *What are your thoughts about continuing with the medicine/treatment?*
● *Have you experienced X (example) side effect?*

Example follow-up questions if the customer expresses low motivation or concern:
● *What do you think might happen if you stop taking the medicine?*
● *What do you need to feel confident with the treatment?*
● *Is there anything that worries you?*

**3. Understand how the practical use of the medicine has worked in daily life**
Example opening questions:
● *How do you feel it has worked taking your medicine?*
● *When do you take your medicine? (time of day/other medicines)*
● *Has it happened that you did not take your medicine on any day?*
 o *What do you think the reason could be?*
 o *Have you thought of any possible solution yourself?*

Example follow-up questions if the customer expresses challenges with adherence:
● *You mentioned that it feels difficult with… what do you think would make it easier?*

**4. Summary**
Encourage the customer to summarise what they take with them and what they will do to remain adherent.
Example question:
● *What do you think is most important to remember to be able to take the medicine daily?*

**Pharmacist's documentation from consultation 2**

**Consultation 2 completed:**Date: Pharmacist:

**Costumer card**

**You have recived a Pharmacy Medicines Consultation with a pharmacist!**

The service includes two consultations with a pharmacist, held 2–4 weeks apart.
● You will receive answers to your questions about your lipid-lowering medicine and the benefits of the treatment.
● You will receive tips and advice to make your medication routine easier in everyday life.

**Things I should keep in mind:**
……………………………………………………………………………………………………………

……………………………………………………………………………………………………………

……………………………………………………………………………………………………………

……………………………………………………………………………………………………………

……………………………………………………………………………………………………………

**Booked consultation:** Visit at the pharmacy Time:
 Phone call Date:
 Video call

Kind regards
Pharmacist and pharmacy:
Contact for rescheduling:
