# Supplementary material for: Improved persistence to statin therapy through a patient counseling intervention in community pharmacies – A nationwide cohort study
Source: Explor Res Clin Soc Pharm. 2025 Dec 22;21:100699. doi: 10.1016/j.rcsop.2025.100699 (PMC12811631; doi:10.1016/j.rcsop.2025.100699)
Supplement: Supplementary file 2 — One-year persistence: intervention vs. non-intervention group in the 127 participating pharmacies. [file mmc2.docx]

**Appendix 2.** One-year persistence: intervention vs. non-intervention group in the 127 participating pharmacies.

**Table A2.1.** *Comparison of persistence after one year between patients who received both consultations (intervention) and those who visited the 127 participating pharmacies and did not receive the intervention
All results are stratified but unadjusted. A total of 227 (1.5%) of all patients in the non-intervention group received only one conversation of the service, and thus did not complete the full intervention as intended per protocol. Information was not available to identify this group and thus exclude them from the analysis.*

|  | **Intervention (I)**  **(n=902)** | | | **Non-intervention (NI)  (n=15104)** | | | **Difference (* = significant)** | | | |
| --- | --- | --- | --- | --- | --- | --- | --- | --- | --- | --- |
|  | **Total** | **Persistent** | | **Total** | **Persistent** | | **%-units** | | **95% C.I.** |  |
|  | **No.** | **No.** | **%** | **No.** | **No.** | **%** |  |  | |  |
| **TOTAL** | 902 | 723 | 80.2 | 15104 | 10993 | 72.8 | 7.4 | (4.7-10.1) | | *** |
| **Sex** |  |  |  |  |  |  |  |  | |  |
| Men | 433 | 341 | 78.8 | 8176 | 6047 | 74.0 | 4.8 | (0.8-8.8) | | *** |
| Women | 469 | 382 | 81.5 | 6928 | 4946 | 71.4 | 10.1 | (6.4-13.7) | | *** |
| **Age** |  |  |  |  |  |  |  |  | |  |
| <55 | 150 | 104 | 69.3 | 3258 | 2135 | 65.5 | 3.8 | (-3.8;11.4) | |  |
| 55-64 | 228 | 183 | 80.3 | 3904 | 2913 | 74.6 | 5.7 | (0.3-11.0) | | *** |
| 65-74 | 303 | 249 | 82.2 | 4318 | 3254 | 75.4 | 6.8 | (2.3-11.3) | | *** |
| ≥75 | 221 | 187 | 84.6 | 3624 | 2691 | 74.3 | 10.3 | (5.4-15.3) | | *** |
| **Level of education** | |  |  |  |  |  |  |  | |  |
| Primary school  (≤9 years) | 131 | 105 | 80.2 | 2774 | 1999 | 72.1 | 8.1 | (1.1-15.1) | | *** |
| Secondary school  (10-12 years) | 418 | 339 | 81.1 | 6837 | 5043 | 73.8 | 7.3 | (3.4-11.2) | | *** |
| Academic (>12 years) | 353 | 279 | 79.0 | 5493 | 3951 | 71.9 | 7.1 | (2.7-11.5) | | *** |
| **Marital status** |  |  |  |  |  |  |  |  | |  |
| Married | 500 | 416 | 83.2 | 8074 | 6035 | 74.8 | 8.4 | (5.0-11.9) | | *** |
| Unmarried | 402 | 307 | 76.4 | 6956 | 4923 | 70.8 | 5.6 | (1.3-9.9) | | *** |
| **Income (SEK)** | |  |  |  |  |  |  |  | |  |
| ≤181,800 | 175 | 135 | 77.1 | 3479 | 2422 | 69.6 | 7.5 | (1.1-13.9) | | *** |
| 181,900-261,300 | 222 | 180 | 81.1 | 3695 | 2679 | 72.5 | 8.6 | (3.2-13.9) | | *** |
| 261,400-382,000 | 264 | 212 | 80.3 | 3902 | 2915 | 74.7 | 5.6 | (0.6-10.6) | | *** |
| >382,000 | 241 | 196 | 81.3 | 3954 | 2942 | 74.4 | 6.9 | (1.8-12.0) | | *** |
| **Country of birth** |  |  |  |  |  |  |  |  | |  |
| Sweden | 794 | 648 | 81.6 | 12110 | 9076 | 80.1 | 1.5 | (-1.3;4.3) | |  |
| Nordic countries excl Sweden | 24 | 22 | 91.7 | 550 | 392 | 71.3 | 20.4 | (8.7-32.1) | | *** |
| EU28 excl Nordic countries | 19 | 14 | 73.7 | 498 | 332 | 66.7 | 7.0 | (-13.2;27.2) | |  |
| Outside the EU28 | 65 | 39 | 60.0 | 1941 | 1191 | 61.4 | -1.4 | (-13.5;10.7) | |  |
| **Prevention status** | |  |  |  |  |  |  |  | |  |
| Secondary prevention | 111 | 90 | 81.1 | 3095 | 2382 | 77.0 | 4.1 | (-3.3;11.6) | |  |
| Primary prevention | 791 | 633 | 80.0 | 12009 | 8611 | 71.7 | 8.3 | (5.4-11.2) | | *** |

*95% confidence interval.*
